# The role of saline irrigation prior to wound closure in the reduction of surgical site infection: a systematic review and meta-analysis

**DOI:** 10.1186/s13037-020-00274-2

**Published:** 2020-12-22

**Authors:** Peter C. Ambe, Tanja Rombey, Julian-Dario Rembe, Johannes Dörner, Hubert Zirngibl, Dawid Pieper

**Affiliations:** 1 Department of General Surgery, Visceral Surgery and Coloproctology, GFO Kliniken Rhein Berg Vinzenz-Pallotti-Hospital Bensberg, Vinzenz-Pallotti-Str. 20, 51429 Bergisch Gladbach, Germany; 2 Department of Surgery, Helios University Hospital Wuppertal, Wuppertal, Germany; 3 Institute for Research in Operative Medicine, Faculty of Health, School of Medicine, Witten/Herdecke University, Witten, Germany

**Keywords:** Abdominal surgery, Normal saline, Surgical site infection, Wound infection, Wound irrigation

## Abstract

**Background:**

Surgical site infection (SSI) describes an infectious complication of surgical wounds. Although SSI is thought to be preventable, it still represents a major cause of morbidity and substantial economic burden on the health system. Wound irrigation (WI) might reduce the level of bacterial contamination, but current data on its role in reducing or preventing SSI is conflicting. Our aim was to investigate the effectiveness of WI with normal saline prior to wound closure for the reduction of SSI in patients undergoing abdominal surgery.

**Methods:**

We conducted a systematic literature search in MEDLINE, EMBASE, and CENTRAL from inception to present, and cross-checked the reference lists of all included primary studies and relevant systematic reviews. (Quasi-) randomized controlled trials (RCTs) investigating the rate of SSI when using normal saline vs. no irrigation prior to wound closure following abdominal surgery were included. Primary outcome was the rate of SSI, secondary outcome the mean length of hospital stay (LOS).

**Results:**

Four RCTs including a total of 1194 patients were included for analysis. All studies compared wound irrigation with normal saline with no wound irrigation prior to wound closure. Their risk of bias was moderate. The relative risk of developing a SSI was lower when wound irrigation with normal saline was performed prior to wound closure although the effect was not statistically significant (risk ratio 0.73, 95%-confidence level: 0.37 to 1.43). Similarly, there was no difference in the LOS amongst both intervention arms.

**Conclusion:**

This systematic review could not identify an advantage for routine irrigation of abdominal wounds with normal saline over no irrigation prior to wound closure in preventing or reducing the rate of SSI.

**Systematic review registration:**

PROSPERO registry number CRD42018082287.

**Supplementary Information:**

The online version contains supplementary material available at 10.1186/s13037-020-00274-2.

## Introduction

Infectious complications represent the most common group of adverse events seen in patients receiving medical care [1]. In surgical disciplines, surgical site infection (SSI), defined as wound infection with microorganisms, especially bacteria, within 30 days following a surgical procedure, represent the most commonly reported complication [2]. The incidence of SSI has been estimated to be as high as 25%, largely depending on the kind of surgery [3]. Thus, SSI constitutes one of the most common nosocomial infections and has been shown to be associated with increased risk of morbidity and mortality, especially in the oncologic setting. Besides, the management of SSI is associated with a substantial financial burden on the health system [4]. The magnitude of this problem is reflected by the existence of numerous guidelines on the prevention of SSI [5–7]. Despite various measures implemented to reduce SSI, like the use of prophylactic single shot antibiotics at the beginning of surgery prior to skin incision, minimal invasive access with less tissue trauma, and the use of wound protectors, the rate of this postoperative complication still remains high [8, 9].

The rationale behind wound irrigation (WI) is to flush the surgical incision with a solution to physically remove cellular debris, trapped fluids and reduce bacterial load. The effect of WI on reducing SSI has been studied before [10–12], but the agents used for WI, the type of surgical procedure and the use of wound protecting devices varied widely in the existing studies [13, 14]. In current guidelines, WI is not addressed as a means of reducing the rate of SSI, probably due to the low level of evidence. Nonetheless, data suggesting a potential benefit of WI prior to skin closure exit [15, 16] and are in line with our clinical experience with routine WI with saline prior wound closure.

This systematic review of randomized controlled trials (RCTs) was undertaken to investigate if WI with normal saline prior to wound closure is effective in reducing SSI in patients undergoing abdominal surgery.

## Materials and methods

The reporting of this systematic review adheres to the Preferred Reporting Items for Systematic Reviews and Meta-Analyses (PRISMA) guidelines [17].

### Protocol and registration

This systematic review was registered in the PROSPERO database (CRD42018082287). Its methods have been previously published in form of a systematic review protocol [18]. No deviations from the protocol occurred.

### Eligibility criteria

RCTs investigating the rate of SSI following WI with normal saline vs. no irrigation prior to wound closure following abdominal surgery were included. Studies were only included if they were published in English or if an English translation was available. All forms of saline wound irrigation with or without the use of a pressure device or syringe were considered eligible.

### Information sources and search

Systematic literature searches were conducted to identify all relevant and eligible published studies. The following bibliographic databases were searched from inception to June 30th 2018: MEDLINE (via PubMed), EMBASE (via EMBASE), and CENTRAL (via the Cochrane library). The MEDLINE search strategy was as follows: (irrigation [tiab] OR “Therapeutic Irrigation”[mesh] OR lavage [tiab]) AND (saline [tiab] OR “Sodium Chloride”[mesh] OR sodium chloride [tiab]) NOT (“Comment” [Publication Type] OR “Letter” [Publication Type] OR “Editorial” [Publication Type]). The reference lists of eligible articles were hand searched by three reviewers (TR, KZ and JDR).

### Study selection process

The title and abstract of each article were screened and assessed against predetermined eligibility criteria by two reviewers (JDR and JD) independently. Subsequently, they assessed the full-texts of all potentially relevant articles and those without an available abstract. Discrepancies were resolved by discussion or by consulting a third reviewer (PCA).

### Data collection process

A data extraction sheet was designed and tested by all investigators. Two reviewers (JDR and JD) independently extracted data from the included studies. Any disagreements were resolved via discussion with PCA and HZ. The extracted data was independently cross-checked by two reviewers (TR and DP).

### Data items

Data regarding the following items was collected: country, study design, setting, dose of saline, number of randomized patients included in the analysis, patient’s demographics (age, sex, body mass index, proportion of patients with diabetes, proportion of smokers), type of surgical procedure and means of access (laparoscopic or open), and perioperative data including procedure-associated information like type of surgery (elective vs. emergency), duration of surgery, use of single-shot antibiotics, and use of wound drains.

Primary outcome was the rate of SSI, secondary outcome the mean length of hospital stay (LOS).

### Risk of bias in individual studies

Two reviewers (DP and TR) independently assessed the risk of bias in the included studies on outcome level using the Cochrane Risk of Bias tool [19]. It consists of six domains, which were judged as having a low, high or unclear risk of bias. All judgments were supported by quoting from or commenting on the studies (see supporting document). Disagreements between the reviewers were resolved through discussion. Information about the risk of bias was incorporated descriptively in the narrative presentation of the results and was considered when grading the quality of evidence.

### Summary measures and synthesis of results

For each outcome reported by two or more studies that was judged to be sufficiently clinically and methodologically homogenous, we performed a meta-analysis using the computer program ReviewManager (RevMan, version 5.3).

For dichotomous outcomes, we calculated relative risks (RRs) using the Mantel-Haenszel [20] method, for continuous outcomes we calculated mean differences (MDs) using the Inverse-Variance (IV) method. For all summary measures, 95% confidence intervals (95% CIs) were calculated. To assess statistical heterogeneity, I^2^ was calculated. I^2^ values of 0 to 40% roughly indicated that heterogeneity might not be important, while higher I^2^ values represented moderate (I^2^ = 30 to 60%), substantial (I^2^ = 50 to 90%) or considerable heterogeneity (I^2^ = 75 to 100%). A random effects model was used to pool the studies’ results and estimate the intervention’s effect if statistical heterogeneity (I^2^ > 50%) was present. In the absence of statistical heterogeneity (I^2^ ≤ 50%), a fixed effects model was used. No additional analyses, such as subgroup or sensitivity analyses, were performed.

### Risk of bias across studies

Since less than 10 studies were included, the risk of publication bias was not formally assessed by creating funnel plots and performing Egger’s test for plot asymmetry [21].

### Confidence in cumulative evidence

The Grading of Recommendations, Assessment, Development and Evaluations (GRADE) approach was used to grade the quality of the evidence (high, moderate, low or very low) from the included studies regarding five domains: risk of bias, indirectness, inconsistency, imprecision and publication bias. Grading was performed with the GRADEpro GDT software by two reviewers (TR and DP) independently. Disagreements between the reviewers were resolved through discussion. Summary of finding tables were created for all outcomes that a meta-analysis was performed for**.**

## Results

### Study selection

**Fig. 1:**
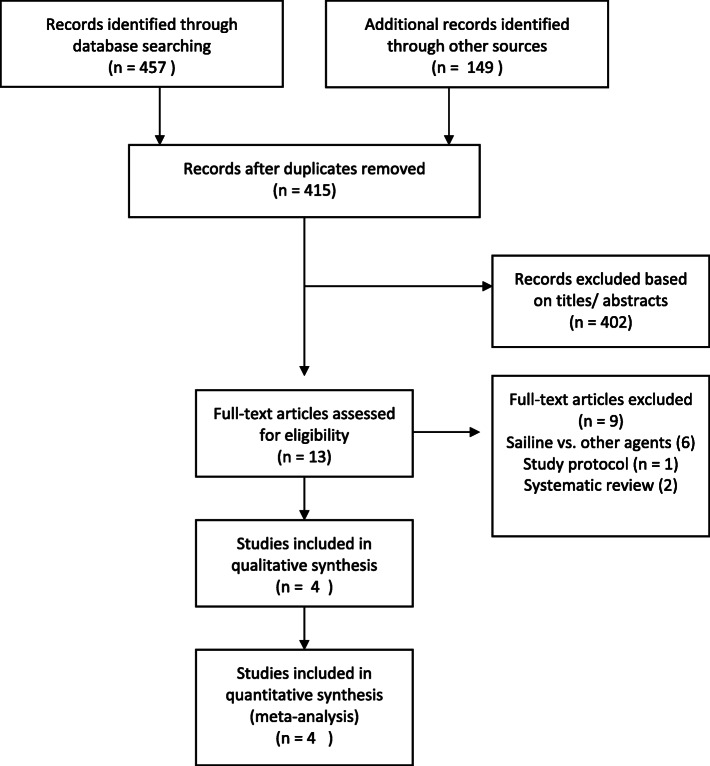
PRISMA diagram

The study selection process and reasons for exclusion are presented in a PRISMA flow diagram, Fig. 1 [22]. The systematic searches retrieved 606 records. After removal of duplicates, 415 records remained, 401 of which were excluded after title and abstract screening. Another nine articles were excluded after full-text assessment [11, 15, 16, 23–28], leaving four eligible (quasi-) RCTs [29–32].

### Study characteristics

The included studies were conducted in Mexico, Jordan and Turkey and were published between 2000 and 2018. In all four studies, an open surgery was performed and prophylactic antibiotics were administered. The studies included between 185 and 520 patients, in total 1194 of whom were analyzed. The patients’ ASA score or immune status was not reported in any study. Mean age ranged from 27 to 40 years. The vast majority of the patients were women, as three of the four studies dealt with gynecological procedures, Table 1.

**Table 1:**
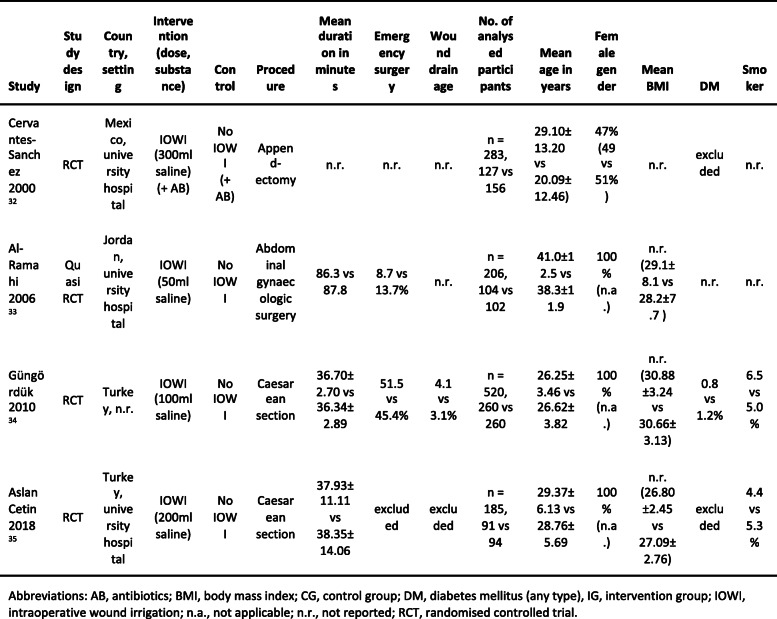
Characteristics of the included studies and participants (intervention vs control group)

### Risk of bias within studies

**Fig. 2:**
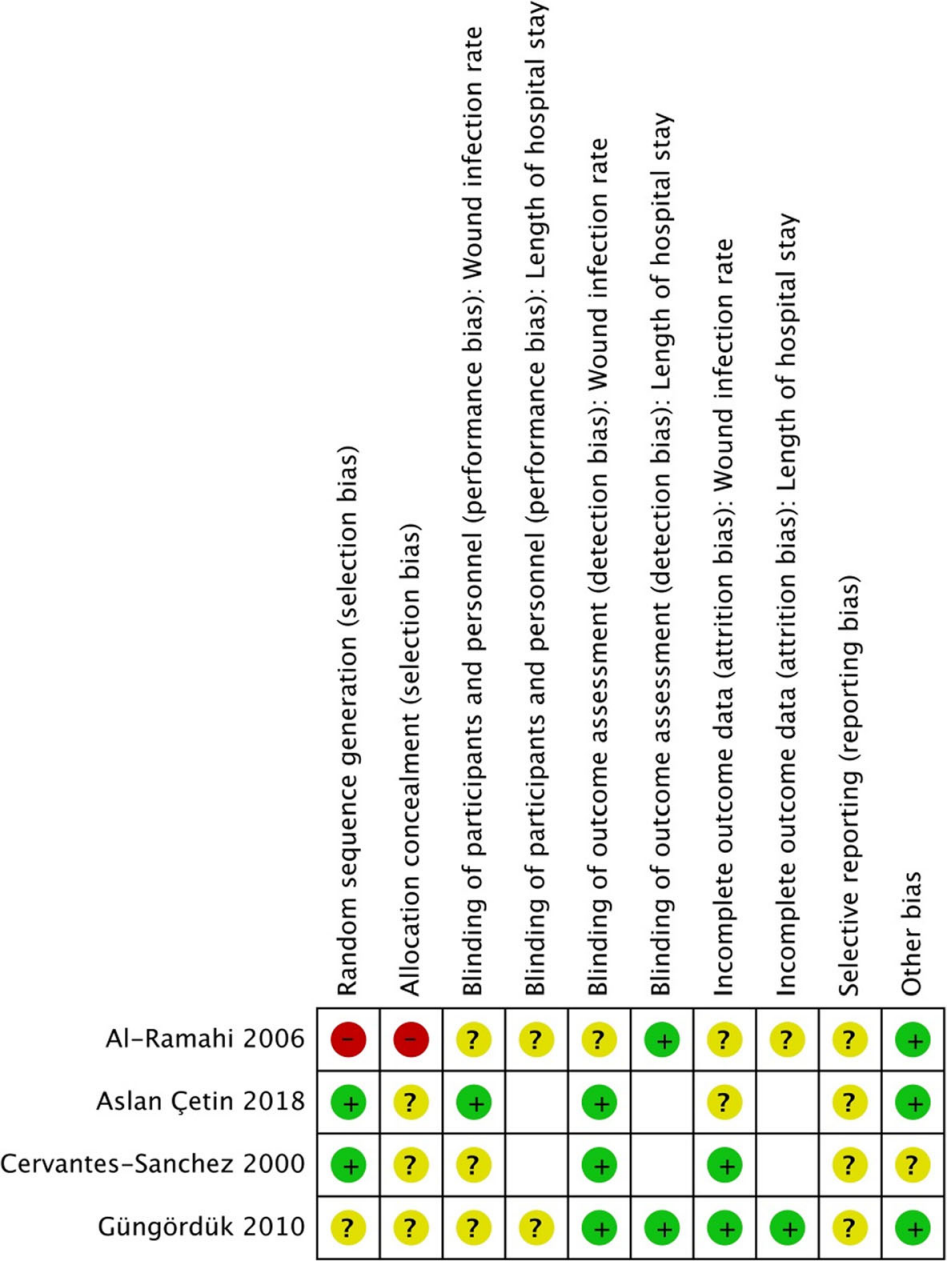
Risk of bias summary

Overall, the risk of bias was unclear for all the studies included, Fig. 2. The risk of selection bias through inadequate random sequence generation was judged to be high in one study that used patient’s hospital number for randomization [30], unclear in a study that did not report how the random sequence was generated [31] and low in the remaining two studies [29, 32]. The risk of selection bias through inadequate allocation concealment was judged to be high in the study using patient’s hospital number for randomization [30] and unclear in the other three studies due to poor reporting [29, 31, 32].

The study personnel was blinded in only one study [32], in which the risk of performance bias was low. The risk of performance bias regarding both outcomes in remaining studies was unclear [29–31]. Apart from one study with an unclear risk of detection bias regarding SSI [30], the risk of detection bias was low in all studies regarding both outcomes [29, 31, 32].

Regarding SSI, the risk of attrition bias was unclear in two studies [30, 32] and low in the other two studies. Regarding the LOS, the risk of attrition bias was unclear [30] or low [31] in the same studies. The risk of selective reporting could not be assessed in any study, as none of them had a published protocol. Except one study with post-hoc exclusion of ineligible patients [29], the studies appeared to be free of other sources bias.

### Results of individual studies and synthesis of results

**Fig. 3:**
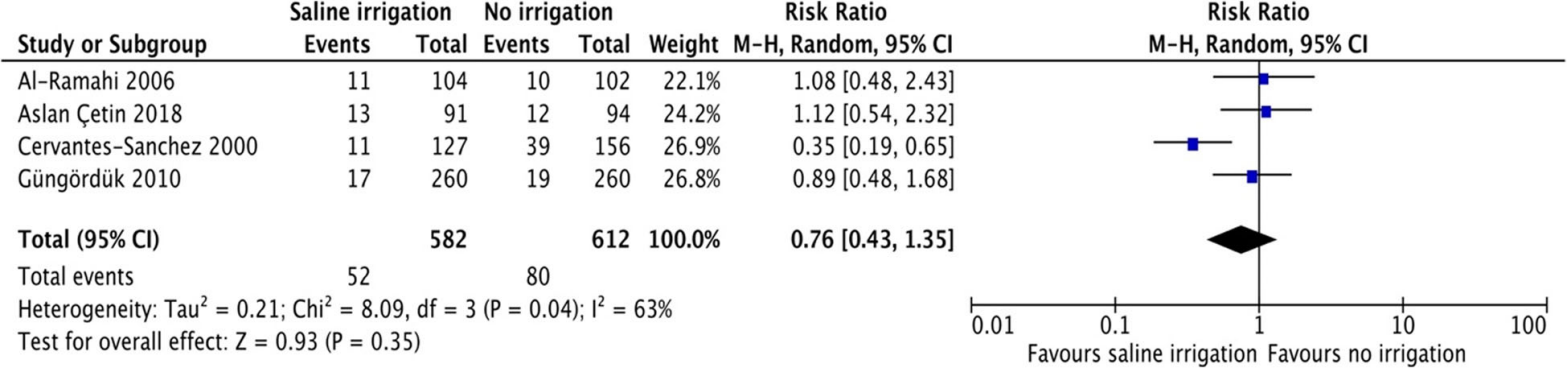
Forest plot of surgical site infection rate

Data regarding the primary outcome, the rate of SSI, was reported by all four studies [29–32]. The overall rate of SSI ranged from 6.9% [31] to 17.6% [29], Table 2. Except for one study [29], there was no difference in the rate of SSI between patients in the intervention group and those in the control group. The pooled RR for SSI following WI with normal saline versus no irrigation prior to closure was 0.76, 95%-CI [0.43 to 1.35], *p* = 0.35, I^2^ = 63%, Fig. 3. Thus pooled data did not show any statistically significant difference amongst both groups with regard to the rate of SSI.

**Table 2:** Results of the included studies per review outcome

	Primary outcome			Secondary outcomes	
Study	SSI rate (IG vs CG)	Relative risk (confidence interval)	***p*** value	Mean hospital LOS (IG vs CG)	***p*** value
Cervantes-Sanchez 2000 [29]	17.6% (8.6 vs 25.0%)*	0.34 (0.13 to 0.61)	0.0006	n.r.	n.r.
Al-Ramahi 2006 [30]	10.2% (10.6 vs 9.8%)	1.08 (n.r.)	n.r.	4.4 vs 4 days	n.r.
Güngördük 2010 [31]	6.9% (6.5 vs 7.3%)	0.88 (0.45 to 1.74)	0.86	2.04 vs 2.05 days	0.67
Aslan Cetin 2018 [32]	13.5% (14.3 vs 12.8%)	1.12 (n.r.)	0.762	n.r.	n.r.

*Abbreviations: CG* control group, *IG* intervention group, *LOS* length of stay, *n.r.* not reported

* indicates statistical significance defined as *p* > 0.05

**Fig. 4:**
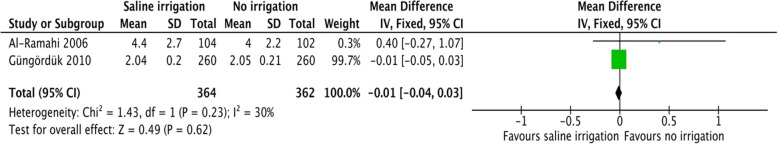
Forest Plot of mean length of hospital stay

None of the studies reported data regarding the rate of re-intervention or re-admission, overall morbidity and mortality, quality of life and resource use. Data regarding the secondary outcome, the mean LOS, was reported by two studies [30, 31], which included a total of 726 patients. The mean LOS did not differ between the intervention and control groups in these studies, Table 2. The pooled mean difference in the mean LOS following WI with normal saline when compared no irrigation prior to wound closure was − 0.01 days, 95%-CI [− 0.04 to 0.03], *p* = 0.62, I^2^ = 30, Fig. 4.

### Confidence in cumulative evidence

The quality of the evidence was judged to be low for both outcomes (see supporting document**)**, which means that our confidence in the effect estimates is limited and the true effect may be substantially different from the estimate of the effect.

## Discussion

The role of routine WI with normal saline vs. no irrigation prior to wound closure in preventing or reducing the rate of SSI following abdominal surgery was investigated in this systematic review. Four RCTs including about 1200 patients were included in the meta-analysis. The primary outcome was the rate of SSI while the mean LOS constituted the second outcome measure. There was no statistically significant difference in the rate of SSI and the mean LOS when WI with normal saline was performed compared to no WI.

A study on the prevalence of nosocomial infections in Germany by Behnke et al. [3] ranked the surgical department behind intensive care unit as the second most common department with a high prevalence of nosocomial infections. The rate of SSI in this study was reported to be as high as 24.3% [3]. This single complication has been shown to be associated with prolonged LOS and increased risk of mortality [33]. Besides, the management of SSI is associated with an overall increase in treatment cost [34].

Wound irrigation so far has been performed in a rather non-standardized manner with different irrigation agents including saline, antibiotics and antiseptics [35]. Thus, current literature on routine WI prior to wound closure is heterogeneous with conflicting findings. This explains the reason why WI has so far not been generally recommended in current guidelines.

A previously published systematic review by Müller et al. from 2015 indicated a significant reduction in SSI following WI [16]. However, studies with various irrigation agents including antibiotics and antiseptics besides normal saline were included in their systematic review. Subgroup analysis indicated that WI with antibiotics significantly reduced the rate of SSI while no advantage was seen following WI with saline. This finding is in accordance with the finding from the present systematic review.

Over 75% of the patients included in this meta-analysis were recruited from three gynecologic studies following caesarean section. Therefore, a vast majority patients included in this systematic review had clean wounds [36]. In fact, there were no significant differences in the rates of SSI amongst patients undergoing WI and those without irrigation in the individual gynecologic studies [30–32]. The remaining study included in this systematic review examined patients undergoing open appendectomy for acute appendicitis. In this group with “clean contaminated wounds”, WI was associated with a significant reduction in the rate of SSI [29]. Therefore, the pooled result of this systematic review can be explained by the heterogeneity of the population included in the meta-analysis.

There was no significant difference amongst both intervention arms with regard to the LOS. However, LOS was reported in only two of the four RCTs included in the meta-analysis [30, 31]. Therefore, this finding might not represent the normal clinical scenario and must be interpreted with caution.

Only RCTs were included in this systematic review with the goal of providing solid evidence for or against routine WI with normal saline prior to wound closure. However, major cofounders that might influence both the rate of SSI and LOS were not systematically reported in the studies included. Therefore, possible effects of cofounders on the predefined outcome measures could not be studies. Lastly, the limited number of studies that met our inclusion criteria can also be considered a limitation to this systematic review.

## Conclusion

Taken together, the findings from this systematic review and meta-analysis including about 1200 patients from four RCTs failed to show any advantage of routine irrigation of abdominal wounds with saline over no irrigation prior to wound closure in reducing SSI. The need for further investigation via well-designed RCTs with larger patient numbers to address the role of WI in preventing SSI cannot be overemphasized.

## Supplementary Information

**Additional file 1.** Summary of findings table.

## Data Availability

The dataset supporting the conclusions of this article is included within the article.
