# Supplementary material for: The role of saline irrigation prior to wound closure in the reduction of surgical site infection: a systematic review and meta-analysis
Source: Patient Saf Surg. 2020 Dec 22;14:47. doi: 10.1186/s13037-020-00274-2 (PMC7756962; doi:10.1186/s13037-020-00274-2)
Supplement: Supplementary file 1 — Additional file 1. Summary of findings table. [file 13037_2020_274_MOESM1_ESM.doc]

**Supplement 1:** Summary of findings table.

| **Saline irrigation compared to no wound irrigation in abdominal surgery** | | | | | |
| --- | --- | --- | --- | --- | --- |
| **Patient or population**: patients undergoing abdominal surgery  **Setting**: hospital  **Intervention**: saline irrigation  **Comparison**: no wound irrigation | | | | | |
| **Outcomes** | **№ of participants (studies) Follow-up** | **Certainty of the evidence (GRADE)** | **Relative effect (95% CI)** | **Anticipated absolute effects** | |
|  |  |  |  | **Risk with no wound irrigation** | **Risk difference with saline irrigation** |
| Surgical site infection rate assessed with: Not specified | 1261 (4 RCTs) | ⨁⨁◯◯ LOW ^a^ | **RR 0.73** (0.37 to 1.43) | 127 per 1.000 | **34 fewer per 1.000** (80 fewer to 55 more) |
| Length of hospital stay | 726 (2 RCTs) | ⨁⨁◯◯ LOW ^b,c^ | *-* | - | MD **0.05 higher** (0.24 lower to 0.34 higher) |
| ***The risk in the intervention group** (and its 95% confidence interval) is based on the assumed risk in the comparison group and the **relative effect** of the intervention (and its 95% CI).  **Abbreviations**  **CI:** Confidence interval; **RR:** Risk ratio; **MD:** Mean difference.  **Explanations**  a. Total number of events is less than 300 and the 95% CI around the pooled estimate of effect includes both, an appreciable benefit and an appreciable harm.  b. I2 = 30%  c. Surrogate outcome. | | | | | |
| **GRADE Working Group grades of evidence** **High certainty:** We are very confident that the true effect lies close to that of the estimate of the effect **Moderate certainty:** We are moderately confident in the effect estimate: The true effect is likely to be close to the estimate of the effect, but there is a possibility that it is substantially different **Low certainty:** Our confidence in the effect estimate is limited: The true effect may be substantially different from the estimate of the effect **Very low certainty:** We have very little confidence in the effect estimate: The true effect is likely to be substantially different from the estimate of effect | | | | | |
